# Early life circadian rhythm disruption in mice alters brain and behavior in adulthood

**DOI:** 10.1038/s41598-022-11335-0

**Published:** 2022-05-05

**Authors:** Rafal W. Ameen, Allison Warshawski, Lucia Fu, Michael C. Antle

**Affiliations:** 1 Department of Psychology, University of Calgary, 2500 University Drive NW, Calgary, AB T2N 1N4 Canada; 2 Hotchkiss Brain Institute, Cummings School of Medicine, University of Calgary, Calgary, AB Canada; 3 Department of Physiology and Pharmacology, Cummings School of Medicine, University of Calgary, Calgary, AB Canada

**Keywords:** Circadian rhythms and sleep, Development of the nervous system, Learning and memory

## Abstract

Healthy sleep supports robust development of the brain and behavior. Modern society presents a host of challenges that can impair and disrupt critical circadian rhythms that reinforce optimal physiological functioning, including the proper timing and consolidation of sleep. While the acute effects of inadequate sleep and disrupted circadian rhythms are being defined, the adverse developmental consequences of disrupted sleep and circadian rhythms are understudied. Here, we exposed mice to disrupting light–dark cycles from birth until weaning and demonstrate that such exposure has adverse impacts on brain and behavior as adults. Mice that experience early-life circadian disruption exhibit more anxiety-like behavior in the elevated plus maze, poorer spatial memory in the Morris Water Maze, and impaired working memory in a delayed match-to-sample task. Additionally, neuron morphology in the amygdala, hippocampus and prefrontal cortex is adversely impacted. Pyramidal cells in these areas had smaller dendritic fields, and pyramidal cells in the prefrontal cortex and hippocampus also exhibited diminished branching orders. Disrupted mice were also hyperactive as adults, but otherwise exhibited no alteration in adult circadian locomotor rhythms. These results highlight that circadian disruption early in life may have long lasting and far-reaching consequences for the development of behavior and the brain.

## Introduction

Infancy and childhood are critical periods for the growth and development of brain and behavior^1^. Adversity during this period can have profound implications for the developmental trajectory, leading to deficits later in life such as increase rates of psychopathology^2,3^. Stress during pregnancy and early life can alter behavior^4^ and brain development^5^. The complex stressor of being raised in a family of lower socioeconomic status^6^ or being raised in a disadvantaged neighbourhood^7^ can adversely affect the developing brain. While low socioeconomic status and disadvantaged neighbourhoods present a host of stressors for the developing child, one area that is consistently adversely impacted in these cases is the quality of sleep^8^.

Infants and children spend a large portion of each 24 h day asleep^9^. As children go through a period of physical, emotional, and psychological growth, they require longer periods of sleep than adults^10–12^. Infancy and childhood are developmental periods that witness tremendous brain maturation, synaptic rearrangements and neural network formation that are critical to support complex cognitive skills^13^. Poor sleep during infancy and childhood are associated with impaired brain development^14–16^, delayed social-emotional development^17–19^, diminished executive functioning^20–22^, delayed language development^23^, lower cognitive performance on neurodevelopmental test^24^ and increased rates of anxiety and depression^25^. Long-term sleep disturbances in childhood can lead to widespread loss of neurons in several brain regions including the hippocampus^26^. As children from disadvantaged neighbourhoods and lower socioeconomic families are at higher risk of sleep problems^8^, it is important to identify and evaluate the various factors that contribute to impaired sleep. One area that may contribute is disruption of circadian rhythms. Circadian rhythms are endogenously generated daily oscillations in behavior and physiology, including the timing and consolidation of sleep. People from certain ethnicities and people of lower socioeconomic status have poorer sleep health^27^, and are more likely to work outside regular daytime work hours^28^. This mismatch between work hours and the body’s normal circadian rhythm is known as social jetlag^29^ and can have profound impact on the health of the worker^30–33^. Chronic circadian disruption in mice can negatively impact their health, leading to impaired immune function and decreased lifespan^34^. Circadian disruption in a worker who is also a caregiver for a young child may impact the circadian health of the child as well. Rodents that experience circadian disruption through exposure to constant light during gestation or the perinatal period exhibit altered behavior as adults^35^, including increased anxiety-like and depression-like behaviors^36,37^, altered locomotor rhythms and circadian responses^38–40^, and impaired hippocampal function^41^. Housing pregnant and nursing mice under a rapidly shifting light–dark (LD) cycle to mimic the circadian disruption of a shift-working parent leads to hyperactivity and impaired social behaviors of their offspring in adulthood^42^. Here, we build upon these studies to examine how circadian disruption with a rapidly shifting LD cycle from birth to weaning can impact anxiety, spatial learning and problem solving in mice as adults. Given that other early-life stresses can alter the adult circadian rhythms of mice^43^, we also explored if adult circadian rhythmicity was altered by exposure to a disrupting light–dark cycle until weaning. Finally, we examined alterations in neuronal complexity within brain areas underlying emotional, spatial and problem-solving behaviors.

## Results

### Rapidly shifting light–dark cycles acutely impair circadian locomotor rhythms

**Figure 1:**
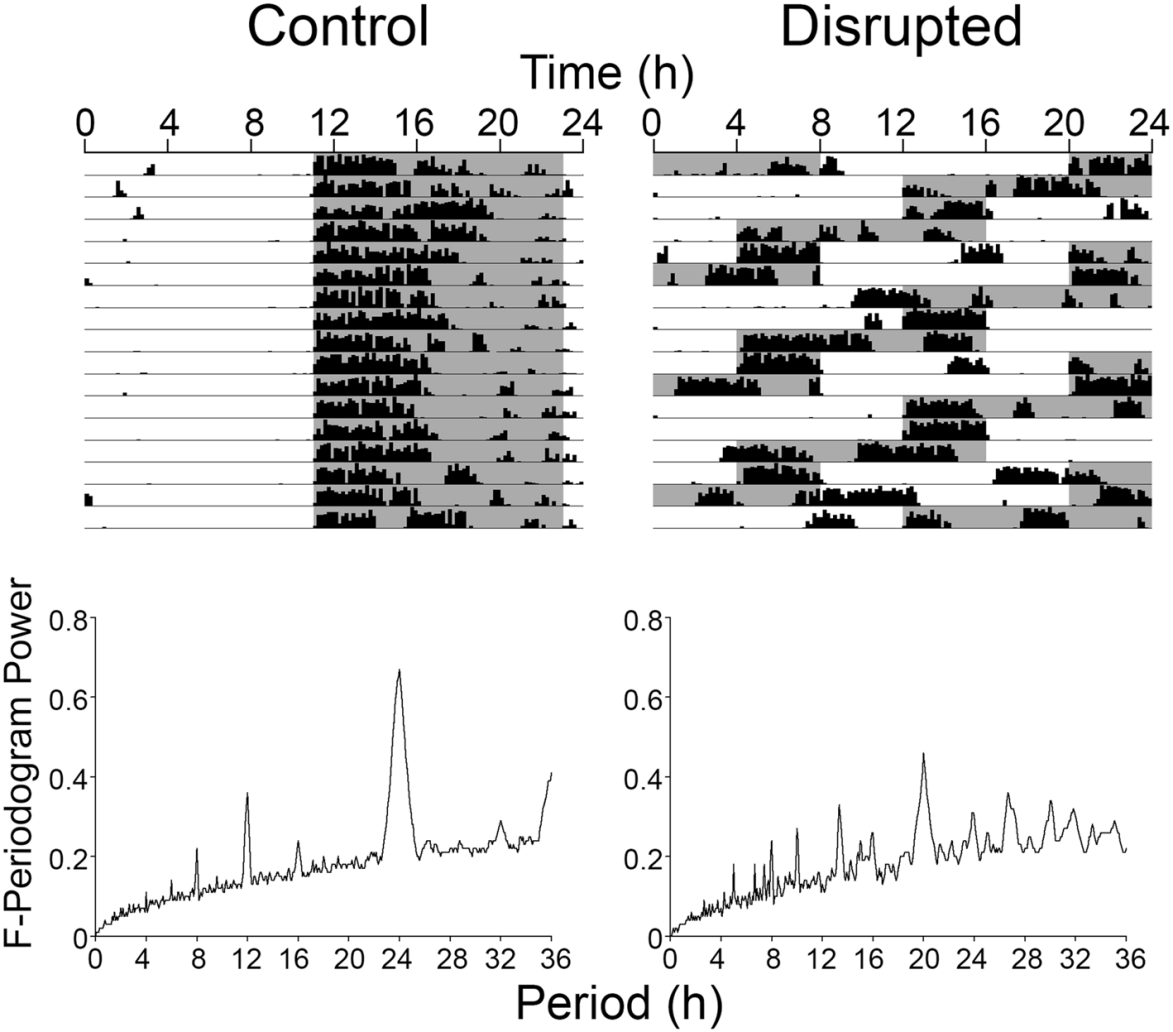
Actograms depicting locomotor activity of adult male mice housed under the control LD cycle (left) or disrupting LD cycle (right) under which the litters were raised from birth until weaning. The graphs are F-periodograms representing the strength of the rhythm at each period.

Beginning on the day of birth, female mice and their litters were exposed to either a regular LD cycle or a disrupting LD cycle where dark onset was advanced by 8 h every second day. To estimate the disrupting effects of such an LD cycle, adult males housed in the same room and exposed to these same LD cycles had their wheel running rhythms recorded (Fig. 1). The strength of the locomotor rhythms was assessed using an F-periodogram analysis. The power at the 24 h period was significantly less (*t*_(2)_ = 8.93, *p* = 0.012) in the disrupted animals (0.31 ± 0.01) than in animals from a regular LD cycle (0.63 ± 0.05). The period with the greatest power in the disrupted animals was at 20 h (0.46 ± 0), and the power at this period was also significantly lower (*t*_(2)_ = 5.00, *p* = 0.038) than that of the peak power observed in control animals at 24 h. This demonstrates that the LD was highly disrupting to the locomotor rhythms in these sample animals with activity frequently occurring during the light phase, and occurring in a fragmented fashion.

### Early-life circadian disruption increases anxiety-like behavior as an adult

**Figure 2:**
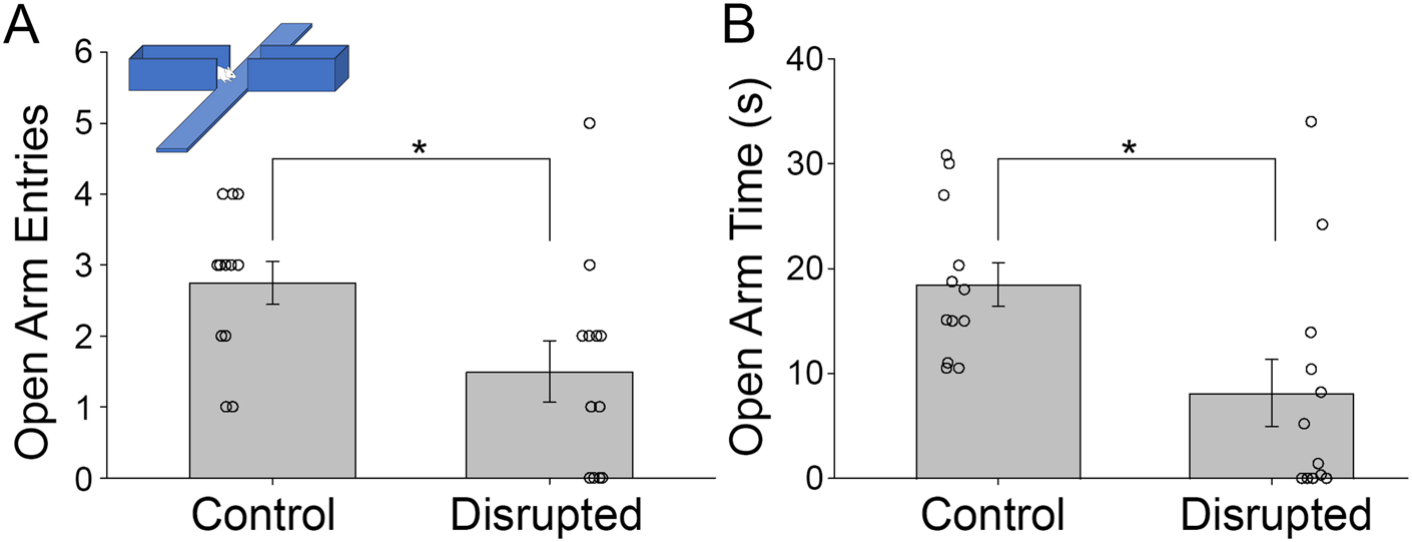
The mean number of entries into the exposed arms of the Elevated Plus Maze (EPM) task by the control and disrupted animals (**A**). The mean time in seconds spent by the control and disrupted groups in the open arms during the EPM task (**B**). The error bars represent the standard error of the mean. The difference between the groups was significant at *p* < 0.05. N = 12/group.

To examine anxiety-like behavior, mice (control n = 12, disrupted n = 12) were assessed using the Elevated Plus Maze (EPM). The disrupted animals had significantly less entries into the open arms (1.5 ± 1.5 entries) than did the undisrupted control group (2.75 ± 1.1 entries, Mann–Whitney *U* = 32.5, n1 = n2 = 12, *p* = 0.02 two-tailed, Fig. 2A). Such diminished exploratory behavior is consistent with higher anxiety-like behavior in the disrupted animals. In addition, the disrupted animals spent significantly less time in the exposed arms (8.1 ± 11.0 s) compared to the controls (18.5 ± 7.3 s Mann–Whitney *U* = 24.0, *n*_1_ = *n*_2_ = 12, *p* = 0.005 two-tailed, Fig. 2B) consistent with higher anxiety-like behavior.

### Early-life circadian disruption impairs spatial navigation as an adult

**Figure 3:**
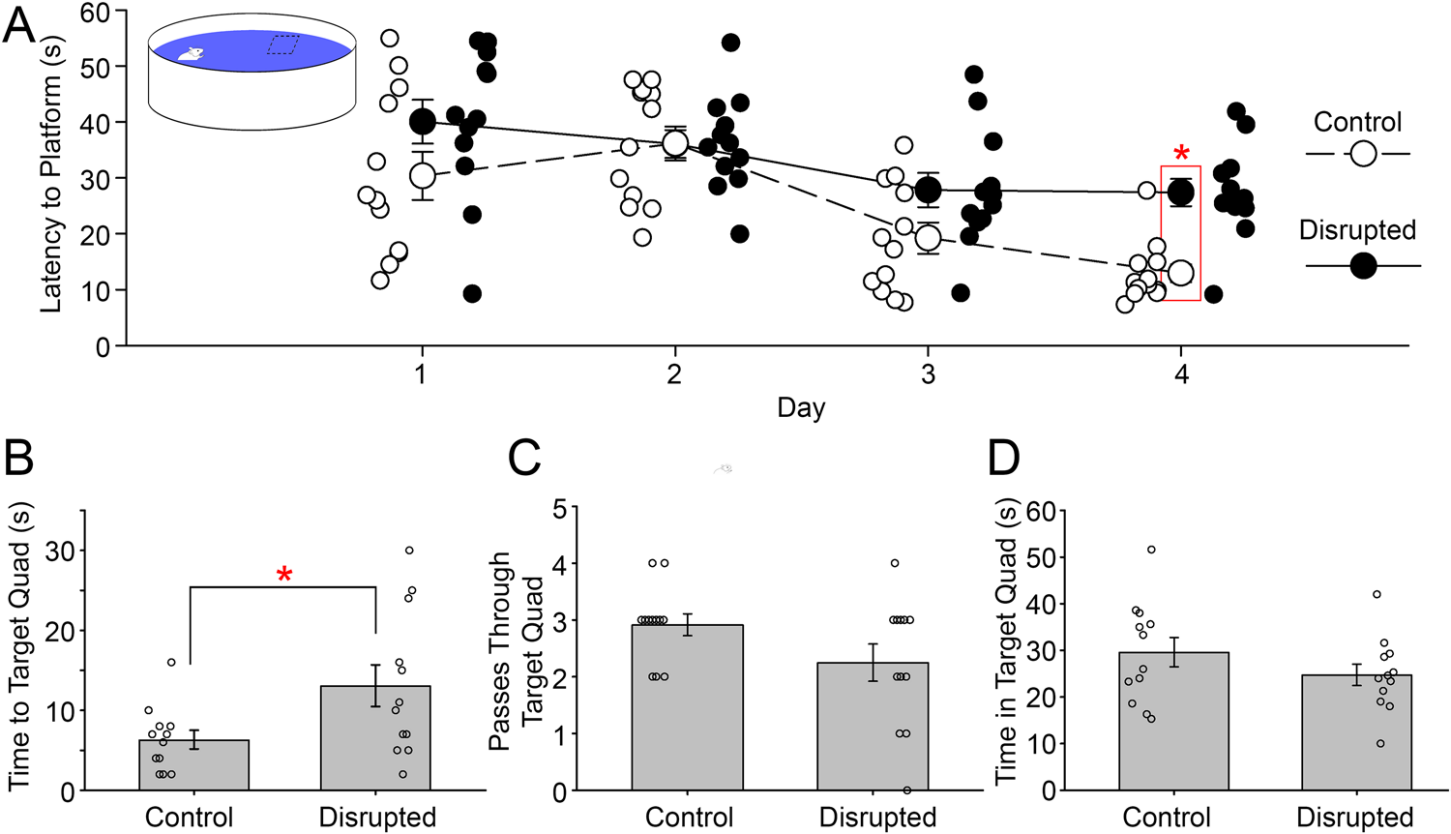
The mean latency, in seconds, to the platform on the four training days of MWM task between the control and disrupted mice (**A**). The latency in seconds to the target quadrant between the control and the disrupted animals on the testing day (day 5) (**B**). The mean number of passing through the target area by the control and disrupted animals on the testing day (day 5) (**C**). The time spent in the target quadrant (quadrant 1) by control and disrupted animals on testing day (day 5) (**D**). The platform was located in quadrant 1 of the apparatus during training. Error bars represent the standard error of the mean. * = *p* < 0.05. N = 12/group.

To examine spatial memory, the same mice were then assessed using Morris Water Maze (MWM). Animals from both groups showed significant improvements in the time they took to locate the platform across the four training days (ANOVA on transformed data, main effect of days, Greenhouse–Geisser, *F*_(2.11, 46.38)_ = 16.01, *p* < 0.001, µ^2^ = 0.42, Fig. 3A). However, the disrupted animals took significantly more time to reach the platform compared to controls over the course of the learning days (ANOVA on transformed data, main effect of groups, *F*_(__1,22)_ = 10.20, *p* = 0.004, µ^2^ = 0.31). While there was no significant interaction between day and group in latency to find the platform (*F*_(3,88)_ = 0.127, *p* = 0.127), planed comparisons revealed a significant pairwise difference on the final day of training. Disrupted animals took significantly longer (13.1 ± 9.1 s) to get to the target quadrant compared to the controls (6.3 ± 4.1 s) on testing day 5 (Independent t-test on transformed data, *t*_(22)_ = 2.35, *p* = 0.028, Fig. 3B). The disrupted animals passed through the target quadrant less often than the controls, but this difference was not statistically significant (Independent t-test on transformed data, *t*_(22)_ = 1.74, *p* = 0.095, *β* = 0.39, Fig. 3C). Moreover, there was no significant difference in the time spent in the target quadrant between disrupted and controls (t_(21)_ = 0.93, *p* = 0.365, *β* = 0.39, Fig. 3D). There was a significant interaction between day and group in swim speed (*F*_(__4,381)_ = 16.9, *p* < 0.001), with the disrupted animals swimming faster (0.15 ± 0.04 m/s) than controls (0.11 ± 0.06 m/s) on day 1 of training and slower (0.12 ± 0.05 m/s) than controls (0.21 ± 0.04 m/s) on day 4 of training. No pairwise differences were observed in swim speed on other training days or on testing day 5.

### Early-life circadian disruption impairs working memory

**Figure 4:**
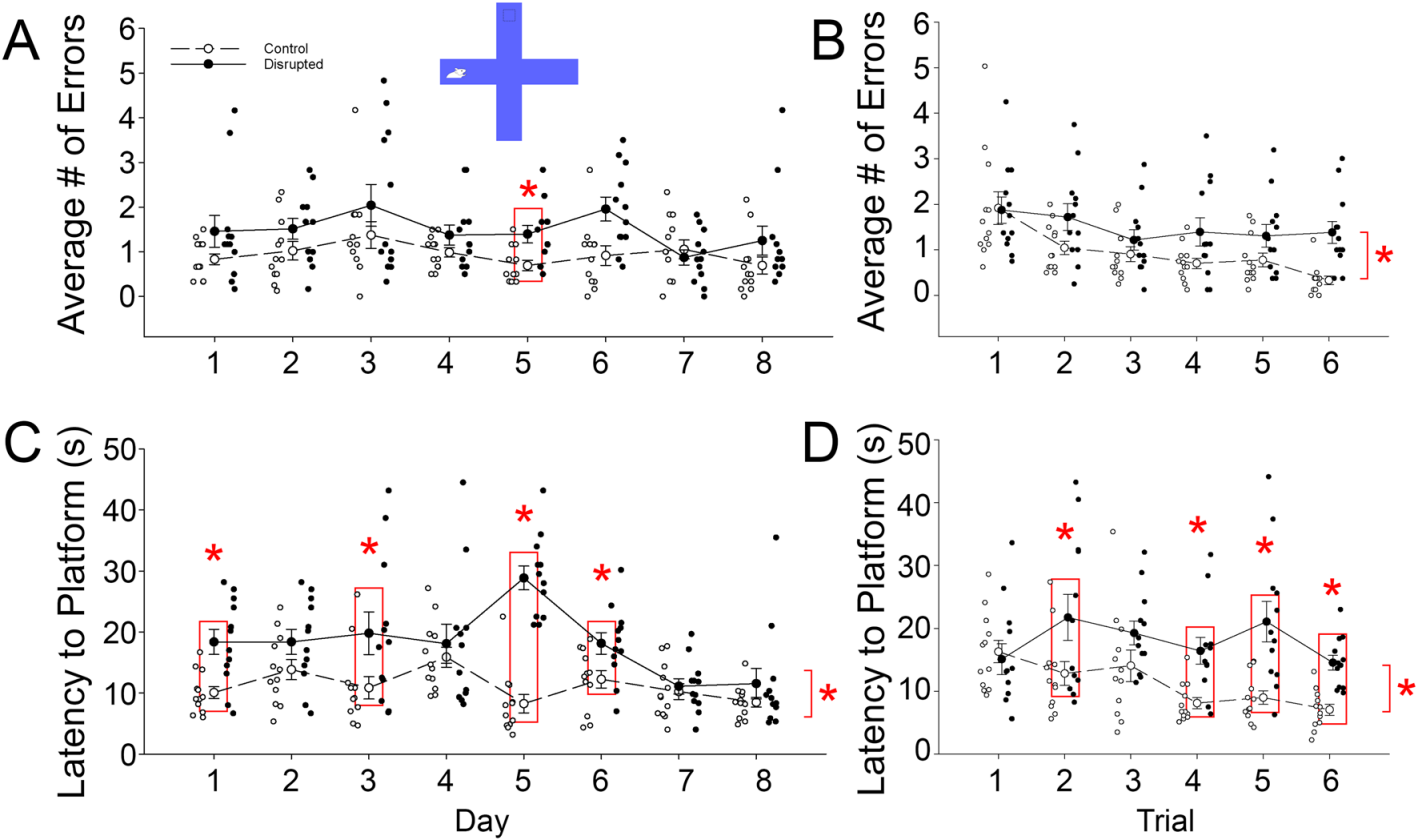
The mean number of entries into the wrong arms between the control and disrupted groups, across the 8 days of the Delayed Match-to-Sample (DMS) task (**A**). Day 5 is statistically significant (*p* < 0.05). The mean number of entries into the wrong arms between the control and disrupted groups, across the 6 trials of the DMS task (**B**). The mean Latency in seconds to the platform on the 8 days of the DMS task between the control and disrupted (**C**), days 1, 3, 5, and 6 are statistically significant (*p* < 0.05). The mean Latency in seconds to the platform on the 6 trials of the DMS task between the control and disrupted (**D**), trials 2, 4, 5, and 6 are statistically significant, (*p* < 0.05). Error bars represent the standard error of the mean. N = 12/group.

There was significant improvement across the days and trials of the test, as indicated by decreased number of wrong entries over time, both within and across days (ANOVA on transformed data, main effect of days *F*_(7,154)_ = 3.33, *p* = 0.009, µ^2^ = 0.13, main effect of trials *F*_(7,154)_ = 7.91, *p* < 0.001, µ^2^ = 0.26, Figs. 4A,B), respectively. Control animals committed significantly fewer errors compared to disrupted animals on Delayed Match-to-Sample (DMS) task (ANOVA on transformed data, between groups effect *F*_(1,22)_ = 17.75, *p* < 0.001, µ^2^ = 0.45).

Performance on the DMS task can also be assessed with latency to find the platform. Animals from control group significantly improved in performance across the days and the trials of the DMS task, in which they became faster in reaching the platform than the disrupted animals over time (ANOVA on transformed data with Greenhouse–Geisser correction, main effect of days *F*_(4.90,07.83)_ = 7.06, *p* < 0.001, µ^2^ = 0.24, main effect of trials *F*_(3.30, 72.53)_ = 2.76, *p* < 0.001, µ^2^ = 0.11, Fig. 4C,D). Disrupted animals took longer to reach the platform across testing days compared to the controls, but their performance showed improvement towards the end of the testing days (ANOVA on transformed data with Greenhouse–Geisser correction, days × groups interaction *F*_(4.90, 107.83)_ = 8.04, *p* < 0.001, µ^2^ = 0.27, trials × groups interaction *F*_(3.3, 72.53)_ = 2.76, *p* = 0.043, µ^2^ = 0.11). In particular, disrupted animals took longer to get to the platform on days 1, 3, 5, and 6 in comparison with the controls (Simple main effect with Holm’s correction, day 1, *F*_(1,23)_ = 12.96, *p* = 0.002; day 3, *F*_(1,23)_ = 5.57, *p* = 0.028; day 5, *F*_(1,23)_ = 77.69, *p* < 0.001; and day 6, *F*_(1,23)_ = 6.23, *p* = 0.021, Fig. 4C). Results from latency to platform across trials showed that disrupted animals and controls did not show differences in performance during the first few trials collapsed across days, but subsequently the disrupted animals performed significantly worse than controls (ANOVA on transformed data with Greenhouse–Geisser correction, trials x groups interaction *F*_(3.3, 72.53)_ = 2.76, *p* = 0.043, µ^2^ = 0.11, Fig. 4D). In particular, disrupted animals took significantly longer time to find the platform on trials 2, 4, 5, and 6 compared to controls (Simple main effect with Holm’s correction, Trial 2 *F*_(1,23)_ = 4.68, *p* = 0.042, Trial 4 F_(1,23)_ = 15.37, *p* = 0.001, Trial 5 *F*_(1,23)_ = 15.39, *p* = 0.001, Trial 6 *F*_(1,23)_ = 26.81, *p* < 0.001).

### Early-life circadian disruption decreases neuronal complexity in the mPFC, dHC, and BLA

**Figure 5:**
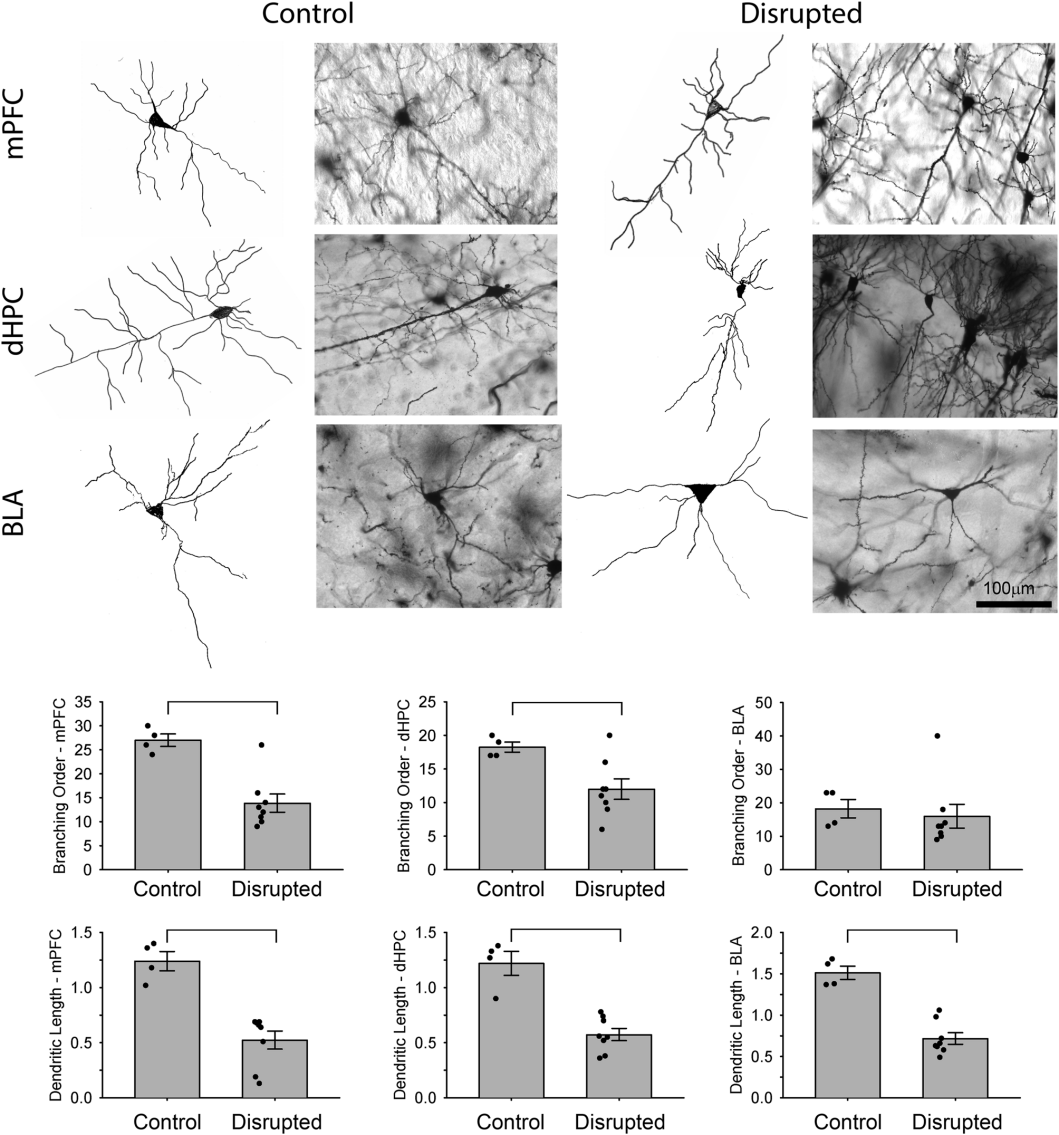
Graphical representation of Golgi-Cox neuroanatomical analyses for disrupted and control animals perfused at P90-110. Neurons of the medial prefrontal cortex (mPFC), dorsal hippocampus (dHC), and basolateral amygdala (BLA) from control and disrupted animals depicted in photomicrographs and camera lucida tracings. The bar graphs represent results from branching order and the dendritic length for control and disrupted animals (*p < 0.001) (3 cells/region/animals were assessed and averaged, n = 4 control, n = 8 disrupted).

When examining neuronal complexity of pyramidal cells, the branching order of the disrupted animals was significantly lower compared to controls in the medial prefrontal cortex (mPFC, Welch’s *F*_(1,10)_ = 20.6, *p* = 0.001, Fig. 5) and in the dorsal hippocampus (dHC, Welch’s *F*_(1,10)_ = 7.6, *p* = 0.02), but was not significantly different from controls in basolateral amygdala (BLA, Welch’s *F*_(1,10)_ = 0.168, *p* = 0.690, *β* = 0.087). When examining dendritic length, animals that experienced early life circadian rhythm disruptions exhibited a significant reduction in dendritic length of the pyramidal neurons in the mPFC (Welch’s *F*_(1,10)_ = 29.1 *p* < 0.001), in the dHC (Welch’s *F*_(1,10)_ = 35.3 *p* < 0.001), and in BLA (Welch’s *F*_(1,10)_ = 47.5, *p* < 0.001, Fig. 5) in comparison with the controls.

### Early-life circadian disruption increases total activity as an adult, but does not alter circadian locomotor rhythms

**Figure 6:**
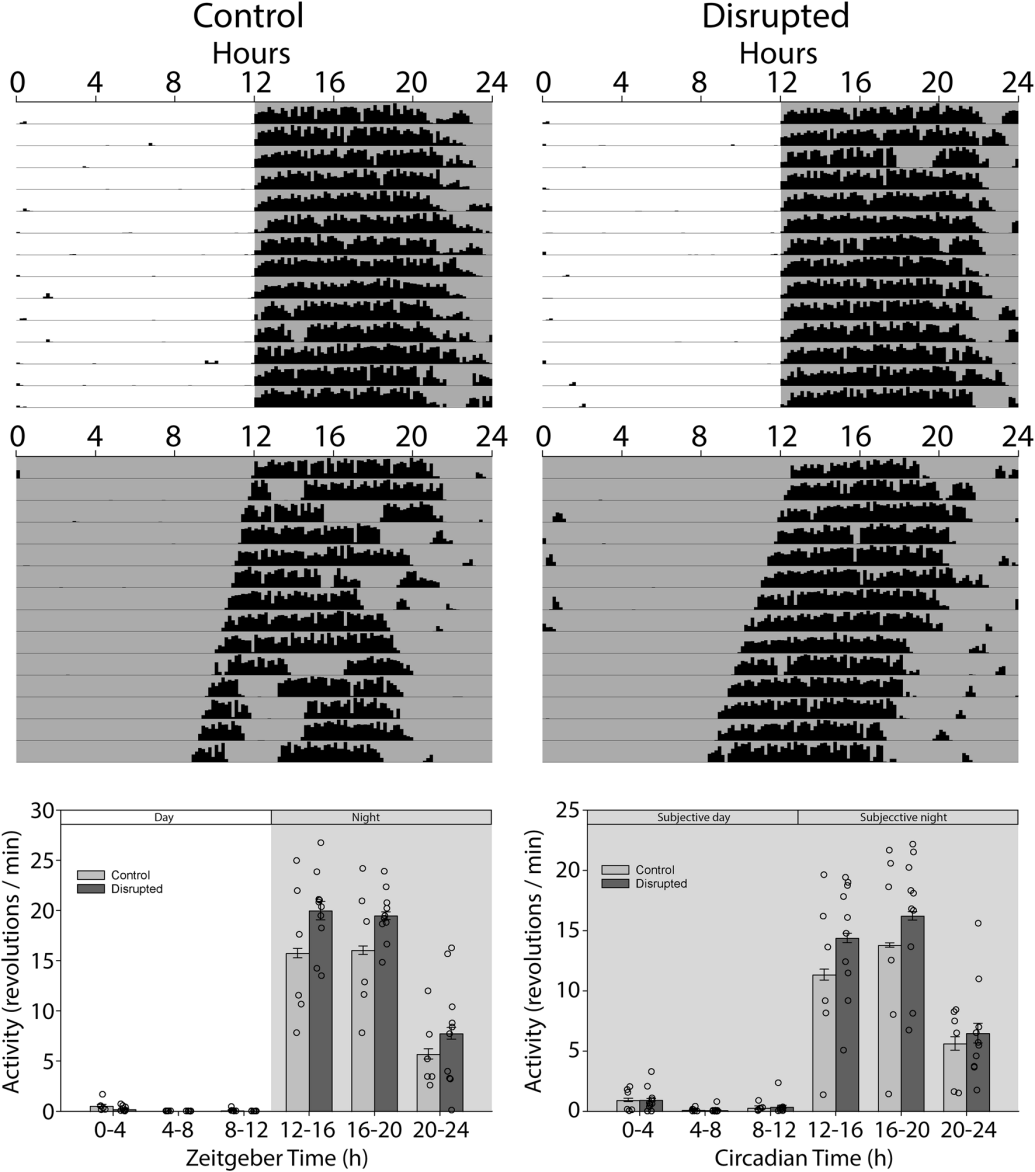
Circadian assessment. Actograms represent wheel running activity of the animals under a light–dark cycle (12–12 LD) and in complete darkness (DD), of controls and disrupted animals. Bar graphs represent activity in 4 h bins during in LD and DD between control and disrupted animals (n = 6 control, n = 10 disrupted).

As adults, the disrupted animals were significantly more active than the control animals when total daily activity was considered (Control 9173 ± 3245 revs/day, Disrupted 11,386 ± 1337 revs/day, *t*_(14)_ = 1.934, one tailed *p* = 0.037). This difference did not carry over to activity levels in constant darkness (DD, Control 7718 ± 3836 revs/day, Disrupted 9255 ± 2853 revs/day, *t*_(14)_ = 0.918, one tailed *p* = 0.18). When activity levels were examined more closely at different circadian phases, there were no significant differences between disrupted and control groups in DD (*F*_(1,70)_ = 0.84, *p* = 0.374, Fig. 6). In LD, while activity at the various night phases was moderately higher in the disrupted animals, this difference was not significant (*F*_(1,70)_ = 3.74, *p* = 0.074) and there was no significant interaction between phase and group (*F*_(5,70)_ = 1.34, *p* = 0.256). In addition, there was no significant difference in the phase angle of entrainment between the control and disrupted groups (*t*_(14)_ = 0.59, *p* = 0.560). There were no differences in the duration of the active phase (alpha) between controls and disrupted animals in either LD (*t*_(14)_ = 0.22, *p* = 0.415) or DD (*t*_(14)_ = 0.38, *p* = 0.384). Free-running period did not differ between groups when assessed with either a χ^2^ periodogram (Controls 23.83 ± 0.18 h, Disrupted 23.78 ± 0.08 h, *t*_(14)_ = 0.767, *p* = 0.456) or when assessed with regression lines fit to activity onsets (Controls 23.83 ± 0.12 h, Disrupted 23.81 ± 0.12 h, *t*_(14)_ = 0.345, *p* = 0.735). In addition, no significant effects were observed between groups for onset variability, mesor, amplitude, acrophase, robustness of the rhythm, or χ^2^ strength, in either LD or DD (Table 1). In most cases, the power for these tests were below *β* = 0.8, so negative results should be interpreted cautiously.

**Table 1:** Assessment of circadian parameters.

Lighting	Measure	Control (M ± SD)	Disrupted (M ± SD)	*p* (*β*)
	N	6	10	
LD	Mesor	64 ± 22	75 ± 10	0.21 (0.24)
	Amplitude	93 ± 32	111 ± 15	0.14 (0.31)
	Acrophase (h)	16.4 ± 0.7	16.6 ± 0.9	0.55 (0.09)
	Onset variability	0.24 ± 0.2	0.17 ± 0.1	0.30 (0.13)
	Phase angle (h)	− 0.24 ± 0.5	− 0.36 ± 0.3	0.56 (0.09)
	Alpha (h)	8.5 ± 1.7	8.7 ± 1.4	0.83 (0.06)
	Robustness (%)	56.9 ± 9.7	48.8 ± 15.7	0.22 (0.23)
DD	Mesor	54 ± 27	65 ± 20	0.38 (0.13)
	Amplitude	77 ± 41	92 ± 29	0.41 (0.12)
	Acrophase	18.4 ± 1.5	17.6 ± 1.1	0.25 (0.20)
	Onset variability	0.41 ± 0.2	0.44 ± 0.4	0.79 (0.05)
	Alpha (h)	9.3 ± 2.5	9.0 ± 1.6	0.77 (0.06)
	Robustness	39.0 ± 20.6	45.6 ± 16.9	0.39 (0.10)
	χ^2^ period (h)	23.8 ± 0.2	23.8 ± 0.1	0.74 (0.11)
	χ^2^ strength	705 ± 275	823 ± 201	0.34 (0.15)

## Discussion

The goal of our study was to investigate the effect of circadian disruptions during postnatal period (PN0-21) on executive functioning, circadian rhythmicity, and neuronal morphology later in life. In particular, we investigated the consequences of early circadian rhythm disturbances on adult spatial learning, working memory, and anxiety. These behaviors depend on the hippocampus, prefrontal cortex, and amygdala, respectively, thus we also examined neuronal complexity in these areas. Our findings demonstrate that early-life circadian disruption affects behavior as an adult, increasing anxiety-like behavior, impairing acquisition and recall in a spatial navigation task and adversely affecting performance on a DMS task that assesses working memory. These changes are mirrored by a decrease in the size and complexity of pyramidal cells in brain areas that underlie each of these behaviors (i.e., BLA for anxiety, dHC for spatial navigation, and mPFC for working memory). While early life circadian disruption leads to some hyperactivity as an adult, no obvious changes to other circadian locomotor rhythm parameters are noted.

Our study used a disrupting light–dark cycle similar to that used by Smarr and colleagues^42^. In that study, hyperactivity and increased anxiety were noted as well. Their study included groups that were exposed to circadian disruption in utero as well as postnatally. In general, in utero disruption led to more severe impairments in adulthood, but significant deficits were still observed when circadian disruption was restricted to the postnatal period, such as was observed here. While Smarr and colleagues^42^ did not observe the same deficits in the EPM that we observed in this study, they did observe an increase in anxiety-like movements while the mice were in the maze. No deficits were noted in that study on a novel object memory task. The two memory tasks that we employed in this study both included spatial components, so the deficit observed in our study may suggest task specific impairments due to early life circadian disruption. Impaired performance on spatial navigation tasks have been observed following in utero circadian disruption by exposure to constant light^41^, suggesting that spatial memory may be more sensitive to early life circadian disruption than visual recognition memory. However, another study in rats failed to note deficits in spatial memory following circadian disruption^36^. In that study, the disrupting constant light exposure only lasted for 1 week that encompassed the late-gestational and early perinatal period, and may not have been sufficient to impair spatial memory abilities, despite being sufficient to increase anxiety-like behaviors.

Alterations in light–dark cycles and light intensity during the postnatal period have been reported to alter properties of circadian rhythms later in life. This can include developmental programming of photoperiod to help prepare the circadian clock to the correct season^44^. Exposure to constant light in the early postnatal period can also alter circadian rhythms in adulthood^38–40,45–47^. In particular, such exposure to constant light appears to make circadian rhythms more robust when re-exposed to constant light as an adult. The circadian assessments in the present study only examined rhythmicity under normal light–dark cycles and constant darkness. Follow-up studies could look at circadian responses under a variety of other paradigms, such as re-entrainment to shifted LD cycles, phase shifts to light pulses, and rhythmicity under other lighting regimens. In particular, given that exposure to constant light early in life leads to stronger rhythmicity under similar conditions as an adult^40,47^, it is possible that the rapidly advancing LD cycle used here could protect the circadian system should it experience similar disrupting light later in life. Additionally, while the rapidly shifting light–dark cycle used in our study clearly disrupted the circadian rhythms of adults, quantifying disruption to the circadian rhythms of the pups themselves during the exposure would be optimal. This might be accomplished if future studies with acoustic rhythms of ultrasonic vocalizations or rustling sounds, infrared motion sensors, telemetry recordings from the mother, or hormonal assays from the pups themselves.

While there was no effect of adult circadian rhythms observed in our study, we did observe an overall increase in locomotor activity in the animals that experienced early life circadian disruption. This replicates and reinforces the observation of Smarr and colleagues^42^ that animals that experienced circadian disruption during gestation and the perinatal period were more hyperactive. Mice that experienced prenatal stress from embryonic days 7–18 also exhibit hyperactivity in wheel running behavior as adults^43^. Gestational exposure of mice to Bisphenol-A also leads to hyperactivity of locomotor rhythms as adults^48^. These findings highlight just how sensitive the developing brain is to a variety of insults that can contribute to hyperactivity later in life.

Early life adversity stress can affect developmental trajectories^4,49^. Disrupting light–dark cycles could be stressful to the mother and pups, leaving open the possibility that some of the results observed here are due to non-specific stress rather than directly due to circadian disruption itself. This hypothesis was tested extensively by Smarr and colleagues^42^ using a disrupting light–dark cycle the same as that used here. Despite their disruption encompassing both the pre and postnatal periods, no canonical stress effects were noted, such as decreased litter size, increase cannibalism, or altered epigenetic modification of genes known to be sensitive to early life stress. If not stress, then this leaves open the possibility that it is the circadian disruption that directly affects development. Given the role that sleep quantity and quality play in brain development^13^, the circadian disruption may have compromised the sleep of the neonatal mice during this critical phase of brain development. Sleep problems have been linked to subsequent changes in brain structures in humans^14–16^. While large scale morphological assessments of brain areas were not conducted here, changes to dendritic length and complexity of pyramidal cells were noted in a number of brain areas that have shown to be diminished in children who previously had poor sleep, including the prefrontal cortex^16^ and the hippocampus^15^.

Previous studies on the adverse effects of circadian disruption in the development of brain and behavior in rodents have emphasized the prenatal^41^ or perinatal^36^ periods. When both have been studied^42^ the effects of disruption during the prenatal period were more severe. Our present results confirm adverse effects of postnatal circadian disruption. However, the first 3 weeks postnatal in mice are comparable to a relatively wide developmental period in humans. Postnatal days 1–10 exhibit developmental stages that correspond to the last trimester of pregnancy in humans, while brain development events in mice at postnatal days 20–21 are roughly equivalent to human brain development events observed at 2–3 years of age in children^50^. This suggests that circadian disruption during the last trimester and extending to the preschool years could still have an impact on brain development. Indeed, poor sleep at 2 years of age is associated with less gray matter at 7 years of age^14^. Additionally, poor sleep at 6–12 months of age^17^, or at 2 years of age^18,19^ were both predictive of delayed social-emotional development in children.

The acute consequences for physical and mental health of circadian disruption are emerging^32,33^, but the findings presented here highlight that early life circadian disruption may be even more insidious, altering development of the brain and behavior, leading to long term deficits that can persist through adulthood. Circadian disruption can alter sleep timing and consolidation, but it may also impact a host of other physiological systems including the digestive, cardiovascular, endocrine, and immune systems^33^. Even the microbiome is sensitive to circadian rhythms^51^ and disruptions here may have far reaching consequences^52^. The manner in which the circadian rhythms of an infant or toddler can be disrupted are pervasive in society, including parents whose work shifts rotate or are outside normal daytime hours, the use of light emitting digital devices, and even social jetlag resulting from early awakenings to accommodate drop offs at childcare for parents who work during regular daytime hours. Future translational work should attempt to quantify circadian rhythms through actigraphy in young children to assess degrees of circadian disruption they may experience under various domestic settings.

## Methods

### Animals

All experimental protocols were approved by the Life and Environmental Sciences Animal Care Committee at the University of Calgary**.** All methods are reported in accordance with ARRIVE guidelines for the reporting of animal experiments. All methods were carried out in accordance with the ethical guidelines and regulations of the Canadian Council on Animal Care. To generate the animals used in this study, we bred adult female C57Bl/6 mice (n = 12), obtained from the University of Calgary Life and Environmental Science Animal Resource Centre breeding colony. Pregnant mothers were randomly assigned to be housed with their litters in a regular 12:12 light–dark (LD) cycle (n = 6) or a disrupting LD cycle (n = 6). Mothers in the regular LD cycle produced 47 pups (n = 20 females, n = 27 males), while mothers in the disrupting LD cycle produced 55 pups (n = 32 females, n = 23 males). A maximum of 2 pups from each litter were used for each separate experiment. Sample sizes used in each experiment were based on sample sizes shown previously to be sufficient to detect meaningful differences in circadian and learning experiments in our lab^43,53,54^. Because circadian rhythms of infant mice in a litter are not easily assessed, additional adult C57Bl/6 male mice (n = 4) were used to assess the acute effects of the disrupting LD cycle (n = 2) on circadian locomotor rhythms relative to that observed in a normal LD cycle (n = 2).

### Early life circadian disruption

On the day of birth (postnatal (PN) day 0), cages with the mothers and their litters assigned to the disrupting LD cycle (6 cages, n = 55 pups) were transferred to the circadian disruption colony room. The 12:12 LD cycle was advanced by 8 h every 2nd day. Litters were exposed to the disrupting LD cycle from birth until weaning (PN0-21). Control litters (6 cages, n = 47 pups) were similarly transported to a room where they were exposed to a stable 12:12 LD cycle, with lights on at 6 a.m. On the day of weaning all animals were transferred back to the main mouse colony room with a regular 12:12 LD cycle (lights on at 6am) and housed with same sex littermates (n = 2–4 animals per cage). Food and water were available ad libitum, and room temperature and humidity were maintained at 21 ± 1 °C and 50% ± 10%, respectively. At the age of 3 months (PN90), animals were assigned to one of three experiments (Behavioral Assessment, Circadian Rhythm Assessment, Golgi-Cox Neuroanatomy), with a maximum of 2 animals per litter contributing to each.

To assess the level of circadian disruptions experienced by animals under the disrupting LD cycle during early life period, adult male mice were placed in cages equipped with wheels and exposed to the disrupted LD schedule (n = 2) or the regular LD cycle (n = 2) in parallel to the mothers and their litters.

### Behavioral assessment

Animals were between PN90-110 days of age at the start of behavioral testing. Two animals, a male and a female, were randomly selected from each litter for the behavioral assessment (n = 12 from each light cycle). On the first day of testing, the animals from both groups were weighed (average weight: control 30.9 ± 6.6 g, disrupted 31.6 ± 6.6 g). The animals were exposed to three different tasks to assess different behaviors. Tasks were presented in the following order over different days: Elevated Plus Maze (EPM, day 1), Morris Water Maze (MWM, days 4–8), Delayed Match-to-Sample (DMS, days 12–19). This order was selected so that the tasks progressed from least to most complex and allowed for anxiety to be assessed by the EPM before the mice had habituated to extensive handling. Animals had a 2–3 day break between each test to minimize any effects that the previous task would have on the subsequent tasks^55^. Performance on each task was tracked using a ceiling-mounted camera centered above the apparatus and connected to HVS Image 2020 Plus software (HVS Image Ltd, Twichenham, Middlesex, UK). Performance was also manually recorded by two observers as a backup in case technical problems hindered the automated data collection.

### Elevated plus maze

The Elevated Plus Maze (EPM) assesses anxiety-like behavior in rodents^56^. Animals were tested in the morning beginning at 9 AM. The EPM suitable for mice (Stoelting Co., Il, USA) was illuminated evenly by an overhead light and 2 side lamps. Mice were placed in the center of the maze facing the open arm and were given 5 min to freely navigate the apparatus. The maze was cleaned with 70% ethanol between each animal.

The percent of time the animal spent in the open arms, the percent of time the animal spent in the closed arms, and the number of entries into open and closed arms were recorded. An arm entry was defined as all four paws of the animal fully within the arm. Animals that made fewer entries into the open arms or spent less time in the open arms were deemed to be more anxious.

### Morris Water Maze

The Morris Water Maze (MWM) is a well-validated task for the study of hippocampal-dependent spatial memory in rodents^57^. A circular white fiberglass pool 1.2 m in diameter with no landmarks on the interior wall was used for this task. The pool was filled with water (19 ± 0.5 °C) to a depth of 20 cm. A clear 12 × 12 cm square platform was placed in the center of a quadrant, 1.5 cm below the surface of the water, and remained in this location throughout all training days. The water was made opaque by adding non-toxic white tempera paint to ensure that the mice could not visually locate the platform in the pool. Several distal cues were placed on the walls of the room. The task was conducted over 5 days in the early part of the light phase (i.e., from about 9 AM to 12 noon) each day. The first 4 days were training days that consisted of 4 trials each day. The fifth day was a single trial testing day with the hidden platform removed (probe trial). For each trial on the training days, the animal was placed into the water facing the pool wall at each of the cardinal compass points (N/E/S/W) in a random order that was the same for each animal that day, but was changed every day. Each animal was given at least 10 min to rest between each trial. Animals were given 60 s to swim around the pool. Once the animal climbed onto the platform, the timer was stopped and the animal was allowed 15 s atop the platform to visually orient itself to its position in the room. Animals that failed to locate the platform within 60 s were gently guided to the platform and were allowed 15 s to orient. Animals were dried and returned to a warm cage to rest until their next trial. The pool was cleaned of any floating particles and the water was stirred between each animal. The time to reach the platform was recorded on each trial.

On the fifth day (probe trial), the platform was removed from the pool and the animal was placed into the water from a novel location opposite of the previous location of the platform. Each animal was given a single trial for a maximum of 60 s to swim freely. For this trial, the initial latency to the platform’s former location, the number of passes through the previous location of the platform, and the time spent in each quadrant were recorded and analyzed.

### Delay Match-To-Sample

Delayed Match-to-Sample (DMS) task assesses working memory in rodents^58,59^. The protocol was adapted from that described by Bimonte-Nelson^59^. The maze consists of four symmetrical walled arms (10 × 32 cm, and 15 cm in depth) arranged in a “plus” shape that were then placed within the MWM pool. A transparent platform (7 × 8 cm) was placed at the end of one arm chosen in a pseudo-random order. The pool was filled with water (19.5 ± 0.5 °C) to a depth of 9 cm. With this depth, the top of the hidden platform was 2 cm below the surface of the water. The spatial cues in the room were replaced and rearranged between the MWM and DMS tasks. Animals were tested over 8 consecutive days, with 6 trials per day during the first half of the light phase (i.e., between 9 A.M. and 12 noon). For each trial, the animal was placed in an arm adjacent to the arm with the platform. The platform remained in the same location through all trials on a given testing day but was moved to a new arm every testing day. Animals were placed into the water facing the end wall of the arm and were allowed to navigate the maze for 90 s. Once the animal located the platform, it was left atop the platform for 15 s to orient to the spatial cues. If the animal entered the arm containing the platform and touched the platform without climbing onto it, the trial was terminated and the animal was gently guided to the platform to begin its platform time. Animals were dried and given at least 5 min to rest in a dry warm cage between trials. The maze was cleaned of any floating particles and the water was stirred between each animal. On subsequent test days the platform was moved to a new arm. For the DMS task, the latency to the platform and the number of entries into the arms without the platform (errors) was recorded.

### Circadian assessment

A different set of animals was used for the circadian assessment (n = 6 controls, n = 10 disrupted LD animals). These animals were 90–120 days old at the start of the circadian assessment. Animals were housed individually in cages equipped with running wheels. A magnetic switch detected the rotation of the wheel, and this was recorded by a computer running Clocklab (Actimetrics Inc. Wilmette, IL, USA). Animals were housed in 12:12 LD for 4 weeks followed by 2 weeks in constant darkness (DD). Activity levels in LD were assessed from days 17–29. An average waveform was created over these days. Activity was divided into six 4 h bins for analysis. Activity levels in DD were assessed over the first 14 days of DD in a similar fashion. Average waveforms were created based on the individual free-running period during these days. Average waveforms were aligned at CT12 between each animal, with CT12 being defined as the first 10 min bin with at least 20% of the maximum activity observed in any 10 min bin that was followed immediately by another such 10 min bin. Activity duration (alpha) in LD and DD were assessed by calculating the time between Clocklab-identified activity onsets on days 17–29 in LD or days 1–14 in DD. Phase angle of entrainment in LD was assessed by comparing the Clocklab-identified time of activity onset in LD on days 17–29 with the time of dark onset. Negative values represented activity onsets that occurred after dark onset. Free-running period was assessed over the first 14 days in DD using two approaches: 1) a χ^2^ periodogram analysis, and 2) by measuring the slope of a regression line fit to the activity onsets. Additional circadian parameters were assessed in LD and DD using a cosinor analysis (R. Refinetti, https://www.circadian.org/ZIP/cosinor.zip) that reports mesor, acrophase, robustness and amplitude. Onset variability in LD and DD was assessed by fitting a regression line to activity onsets in Clocklab 6.1.02 and using the reported error term as a variability measure. Quality of the rhythms was also assessed using the χ^2^ statistic reported in Clocklab, which is a least-squares goodness-of-fit statistic. Rhythms with highly stable outputs will have a larger χ^2^ statistic, while rhythms that are variable and unstable day-to-day will have low χ^2^ statistics.

Adult males were recorded for 30 days in parallel to the litters during either normal (n = 2) or disrupting LD (n = 2). Their locomotor rhythms were assessed using Clocklab’s F-Periodogram routine.

### Neuron morphology

A different set of animals (n = 4 control LD, n = 8 disrupted LD) was used for the Golgi-Cox histological assessment. At PN90 animals were rendered unconscious with isoflurane and were then given an overdose of sodium pentobarbital (Euthanyl, ip, 500 mg/kg). They were then perfused with 0.9% NaCl. Brains were extracted and processed using the protocol described by Zaqout and Kaindl^60^. Briefly, brains were immersed in Golgi-Cox solution for 10 days in darkness at room temperature. Brains were then transferred into tissue protectant where they were stored for 2–3 days in 4 °C temperature in complete darkness. The brains were then sliced using vibratome at 60 Hz and thickness of 200 µm, and the sections were mounted onto gelatin coated slides. At the end of the process, the slides were left to dry for 4–5 days in the dark. Slides were then dehydrated and developed according to the protocol detailed by Gibb and Kolb^61^. The slides were then coverslipped with 10 drops of Eukitt solution (Fluka Analytical, Germany). Finally, slides were sealed with nail polish and were left flat to dry in the dark for 48 h before imaging.

For each animal, researchers blinded to the treatment conditions traced 4 pyramidal cells from each of 3 regions of the brain: the mPFC, the CA3 region of the dHC, and the BLA. Tracings were conducted using the 40× objective on an Olympus BX-51 microscope equipped with a camera Lucida tracing arm. The traced neurons selected were well impregnated and not obscured by astrocytes or large clusters of neighboring dendrites. In addition, the apical and the basilar dendrites of the traced neurons were intact and visible in the plane of section. Dendrite length was calculated using Scholl analysis as described by Christensen and colleagues^62^. Complexity of neurons was assessed using the branching order approach described by Coleman and Reisen^63^. The branching order was measured from both the apical and the basilar dendrites. Two researchers blinded to the treatment assessed each tracing, and the average of the two assessments was used for analysis unless large differences in assessments were calculated, in which case the individual assessments were redone.

### Statistical analysis

Statistical analysis was carried out in SPSS version 26 and SigmaPlot 14.5. To study anxiety-like behavior of the disrupted mice compared to the controls, non-parametric Mann–Whitney U tests were conducted to study entries into, and time spent in, open arms. Latency to the hidden platform on training days of the MWM task was analyzed using a 2 (Groups: disrupted and control animals) × 4 (Days of training) × 4 (Trials) factorial ANOVA. To test MWM spatial memory performance on the probe day (day 5), independent samples t-tests were conducted to analyze latency to the previously located platform, the number of passes made through the previous platform location, and the time spent in the target quadrant. To examine performance in working memory in the DMS task, the latency to platform and the mean number of errors (i.e., entries into the arms other than the target arm with the platform) were analyzed using 2 (groups: disrupted, and control) × 6 (trials per day) × 8 (experiment day) factorial ANOVAs. For the circadian data, a two-way repeated measures ANOVA was conducted to study differences in total activity in light–dark condition (12–12 LD cycle) and in complete darkness (DD). Independent samples t-tests were used to test for differences in alpha in LD and DD, phase angle of entrainment in LD, and free-running period in DD. In cases where homogeneity of variance was not achieved, the non-parametric Mann–Whitney U test was used. Due to the unequal sample sizes, Welch’s F tests were used to analyze dendritic length and dendritic complexity in the Golgi-Cox analysis. Prior to conducting the ANOVA tests, the assumptions of normality, sphericity, and homogeneity of the variance were assessed. In the case where homogeneity of variance was not achieved, a square root transformation was applied to the data prior to running the ANOVA. Square root transformation was used to reduce the variability in the data in an unbiased manner. In cases where sphericity was not achieved, the Greenhouse–Geisser correction was employed. Significant interactions were followed up with pairwise comparisons using the Holm’s correction for multiple comparisons with alpha set at 0.05.
